# Divergent Floral and Vegetative Trait Responses to Abiotic Factors Drive Phenotypic Diversity in *Platanthera dilatata* (Orchidaceae) Across Alaska

**DOI:** 10.1002/ece3.71594

**Published:** 2025-06-21

**Authors:** Marlin L. Bowles, Lisa E. Wallace

**Affiliations:** ^1^ The Morton Arboretum (Retired) Lisle Illinois USA; ^2^ Department of Biological Sciences Old Dominion University Norfolk Virginia USA

**Keywords:** Alaska, Berg hypothesis, climate, digital herbarium specimens, flower size, plant size, *Platanthera dilatata*, pollinator‐mediated selection, spur length

## Abstract

Multiple traits make up an integrated phenotype, but traits may be shaped by differing drivers. It is often assumed that selection by biotic agents drives floral variation, but abiotic factors are also known to influence floral traits. Understanding trait covariations and their association with diverse environmental factors is a central question in evolution and ecology. Here, we studied covariation in floral and vegetative traits in relation to climate and topo‐edaphic variation in the orchid *Platanthera dilatata* . In *Platanthera*, nectar spur length is associated with pollinator tongue length, but much variation exists in other traits too, likely to be influenced by abiotic factors. We analyzed floral, inflorescence, and leaf measures from 146 digital herbarium specimens of *P. dilatata* collected in Alaska, USA, and nearby areas, asking whether phenotypic variation occurred across regions and habitats and how abiotic factors affect these patterns. Our results suggest a continuum of multi‐trait variation in *P. dilatata* and suggest that individual traits may be under contrasting influence by the regional climate, local topo‐edaphic conditions, and pollinator‐mediated selection. Phenotypes were distributed among geographic regions, as well as habitats. Plants with larger leaves, inflorescences, sepals, and lips tended to predominate in coastal meadows with moderate climates, while those with smaller traits tended to predominate in continental wet meadows with extreme climates, as well as fens and muskeg bogs. Nectar spur length was poorly correlated with leaf, inflorescence, and flower size and had lower correlations with climatic factors, suggesting that spurs respond to other factors. Differentiating among varieties by spur–lip length relationships may be problematic because of continuous independent variation in both traits. Understanding the mechanisms that generate phenotypic variation in *P. dilatata* would benefit from studies of the underlying genetic control, propensity for phenotypic plasticity across traits, and historical perspectives on the relationships of populations with similar phenotypes.

## Graphical abstract

The study of trait covariations offers insights into the origins of phenotypic variation. In *Platanthera dilatata* , nectar spur length varies independently of leaf, inflorescence, and flower size, suggesting that these traits independently respond to differing environmental pressures.

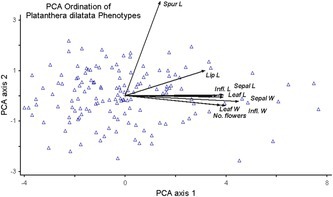

## 1 Introduction

The integrated phenotype of an organism reflects the correlated structure and function of individual traits in response to abiotic and biotic influence (Murren 2002, 2012). Traits may be correlated or uncorrelated (Berg 1960), depending on the direction of these factors and underlying developmental constraints. For example, floral traits are thought to respond to pollinator‐mediated selection, but variation in vegetative traits may reflect stronger responses to abiotic factors, which may explain why ecotypes can be associated with different habitats. Understanding the influence of different external and internal factors on the integrated phenotype and the resulting effect on the distribution of intraspecific phenotypic variation is of great interest in functional ecology and evolution (Albert et al. 2011; Helsen et al. 2017; Messier et al. 2010; Murren 2012).

The manifestation of geographically structured phenotypic groups represents a complex interaction involving both abiotic and biotic factors. The latter may include selection by pollinators and other biotic agents that affect reproduction and survivorship and has often been tied to the extensive floral variation observable in angiosperms (Caruso et al. 2018; Sletvold 2019). Nevertheless, abiotic factors are increasingly recognized to also have an influence on floral traits (Arista et al. 2013; Caruso et al. 2018, 2020) and drive variation in vegetative traits (Helsen et al. 2017). Contrasting selection pressures and variable strength could lead to independent responses by traits, particularly within flowers, and ultimately generate intraspecific variation (Romero‐Bravo and Castellanos 2024). Phenotypic variation can indicate adaptive potential (Geber and Griffen 2003), and it can lead to diversification and speciation across scales of space and time.

Climatic and topo‐edaphic factors represent primary abiotic factors affecting the distribution of organisms (Meier et al. 2010). Climatic factors vary across major climate regimes in relation to geographic features such as coastal and continental landmasses, as well as along latitudinal or elevational gradients. Climatic variables include temperature and precipitation means and extremes, as well as their seasonality and diurnal variability, which can cause increases or decreases in physiological constraints on species growth and distribution (O'Donnell and Ignizio 2012), resulting in corresponding phenotypic patterns (Kuppler et al. 2020). Phenotypic plasticity often varies along climatic gradients in response to physiological stress induced by extreme conditions, such as colder temperatures; greater variation is associated with reduced stress, but, conversely, reduced morphology and physiology are associated with increased stress (Gratani et al. 2012; Hulshof et al. 2013; Stotz et al. 2021). Elevational gradients can drive local climatic extremes and environmental stress gradients, leading to corresponding phenotypic trait responses such as smaller stature, especially in alpine conditions (Gentili et al. 2013). Likewise, there is evidence for genetic differentiation among phenotypes along elevational gradients (Halbritter et al. 2018).

In Orchidaceae, many *Platanthera* species exhibit substantial floral phenotypic variation, frequently attributed to pollinator‐mediated selection (Hapeman and Inoue 1997), but there is also considerable variation in vegetative traits that suggests other factors must also shape the integrated phenotypes within species. At larger geographic scales, where pollinators reach their distributional limits, selection by pollinators is a reasonable hypothesis for intraspecific variation in certain floral traits. However, as environmental factors that drive intraspecific population differentiation often operate at smaller spatial scales (Huang et al. 2024; Wallace and Bowles 2023), it seems less likely that floral variation can be maintained solely by pollinator‐mediated selection, and other factors must also be considered for the origin and maintenance of integrated phenotypic expression within species.

As vegetative traits tend to be more sensitive to environmental variation than specialized floral traits selected by pollinators, decoupling these traits is critical for understanding how different evolutionary forces shape plant form (Berg 1960; Armbruster et al. 1999; Pélabon et al. 2013; Gibert et al. 2025). Covariation of some floral and vegetative traits is to be expected because floral parts are derived from leaves and still share components of their genetic systems (Armbruster et al. 1999). Floral traits also differ in the relative strengths of their responses to pollinator selection, where spur length, which affects pollinator efficiency, can be significantly stronger than floral size, which affects pollinator attraction (Boberg and Ågren 2009; Caruso et al. 2018; Sletvold 2019). Spur length may be under strong pollinator‐mediated selection in *Platanthera* (Robertson and Wyatt 1990; Hapeman and Inoue 1997) and may covary less with other floral parts and vegetative traits (e.g., Bateman et al. 2023).

In this paper, we examine the distribution of phenotypic variation of *Platanthera dilatata* (Pursh) Lindl. Ex L.C. Beck across Alaska using vegetative and floral trait metrics from digitized herbarium specimens. Numerous studies have quantified floral variation in *P. dilatata* populations from diverse areas of its range (Wallace 2003; Adhikari and Wallace 2014; Wallace and Bowles 2023; Plendi et al. 2024), but phenotypic expression that integrates both vegetative and floral traits has not been investigated in *P. dilatata* or in association with a wide range of abiotic conditions and habitats. *P. dilatata* has flowers that exhibit varying spur lengths, which serve as the diagnostic trait to delimit varieties: var. *albiflora* has short spurs relative to lip length, var. *dilatata* has spurs similar to lip length, and var. *leucostachys* has long spurs relative to lip length (Sheviak 2002). Local variation in floral phenotypes has repeatedly been found. For example, in Southeast Alaska, large‐ and small‐flowered phenotypes of *P. dilatata* differ genetically among habitats, and those habitats supporting smaller‐flowered plants may reflect resource limitation or stress (Wallace and Bowles 2023). Likewise, flower size of *P. dilatata* decreased along an increasing elevational gradient in adjacent British Columbia, which may reflect resource limitation (Plendi et al. 2024). In this study, we compared variation in vegetative and floral traits and their distributions among geographic regions and habitats that support a wide range in climatic and edaphic conditions to answer the following questions:

1. How is phenotypic variation structured in association with regions and habitats and their effects on climate and edaphic conditions?
2. Are floral and non‐floral traits decoupled, with reduced phenotypic correlation and contrasting responses to abiotic factors?
3. How does the observed phenotypic variation of *P. dilatata* in Alaska correspond to infraspecific varietal designations?

## 2 Materials and Methods

### 2.1 Study Species

*P. dilatata* has inflorescences of small (6–15 mm long) white flowers; the number of flowers per inflorescence can range from fewer than 20 flowers to more than 75 flowers (Luer 1975; Sheviak 2002). *P. dilatata* appears to be dependent upon insect pollination for seed production (Sheviak 1999), and seed pod production averaged > 80% across three sites (*N* = 16 inflorescences) in Southeast Alaska (M. L. Bowles, unpublished data). Nectar is produced in a floral spur accessed by pollinators; floral scent ranges from spicy to musky and may be released either nocturnally or diurnally (Sheviak 2002; Argue 2012). Column structure positions viscidia parallel on either side of the nectar spur opening, apparently adapted for attachment to the proboscis of a visiting insect (Argue 2012). While floral variation is the most studied aspect of this species, there is also variation in vegetative traits, including plant height, leaf number, leaf shape, and inflorescence size (Sheviak 2002).

### 2.2 Study Area

**FIGURE 1:**
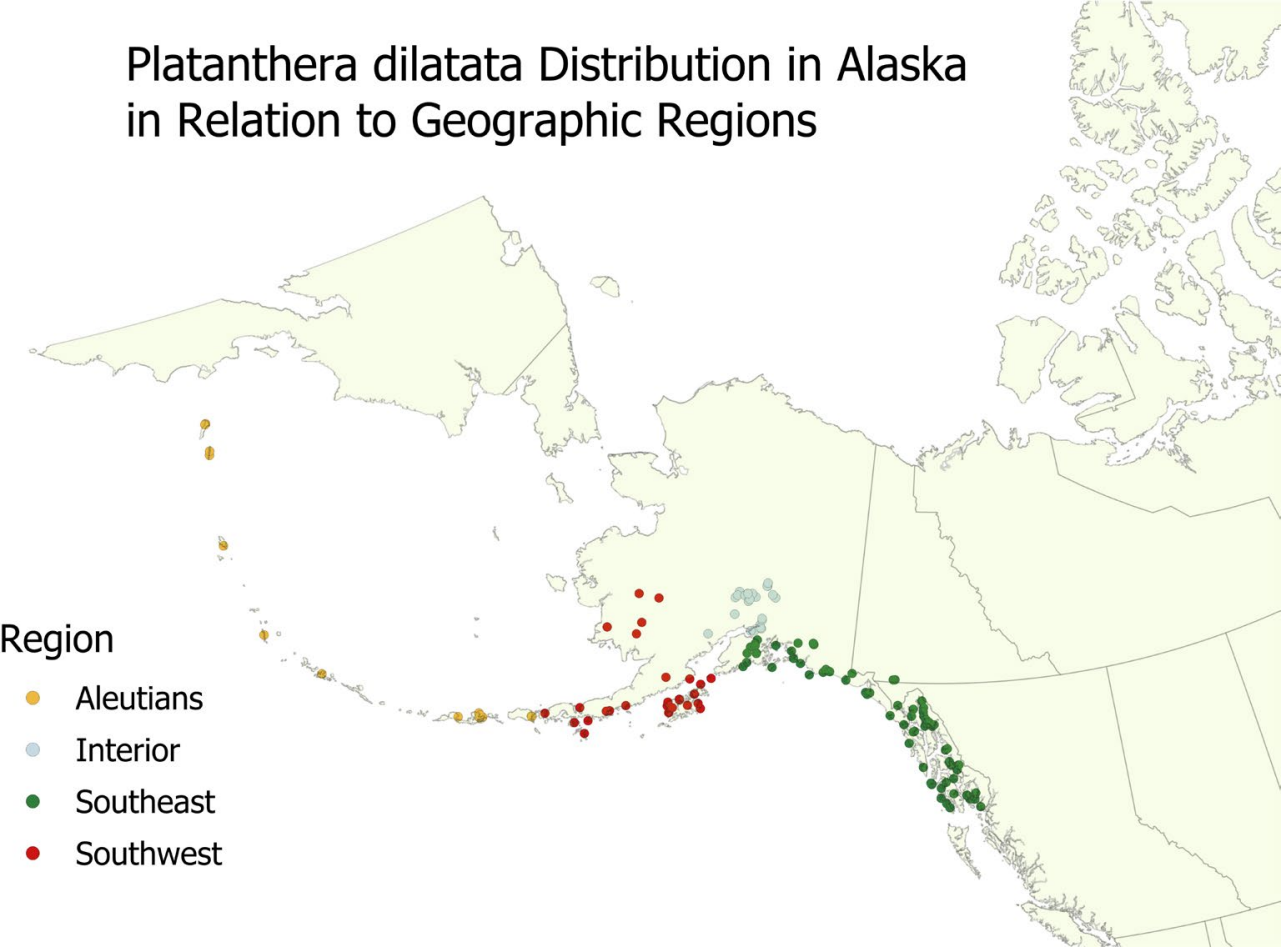
Specimen locations in relation to geographic regions. Regional boundaries are mapped in Andres (1999).

*P. dilatata* is distributed across the northern U.S. and Canada, reaching as far south as New Mexico and as far north as Alaska. Northwest North America may be the center of variability in *P. dilatata* (Sheviak 2002). Populations occur across the longitudinal breadth of Alaska, extending about 3000 km from the western Aleutian Islands to Southeast Alaska and about 1000 km latitudinally from south coastal to interior Alaska. This distribution encompasses multiple geographic regions (Figure 1), including the Pacific Rainforest of coastal southeast and south‐central Alaska, the interior forest of central Alaska, Southwest Alaska, the Alaska Peninsula, and the Aleutian Islands (Hultén 1968; Andres 1999; Figure 1). For this study, we refer to these four regions as Southeast Alaska, Interior Alaska, Southwest Alaska, and the Aleutian Islands, respectively. Climate variation among these regions is controlled by their proximity to the marine environment and the continental landmass (Bienek et al. 2012). The Aleutian Islands and coastal Southeast and Southwest Alaska have relatively mild winters and cool summers, while interior Alaska has colder winters and warm summers.

Based on specimen habitat descriptions and published literature, we apply data analysis to four predominant vegetation habitats occupied by *P. dilatata* : herbaceous meadow, wet meadow, fen, and muskeg bog, which range in elevation from sea level to alpine (Hultén 1968; Stone 1993; Shephard 1995; Boggs 2000; Talbot and Talbot 2010; Ping et al. 2017; Wallace and Bowles 2023). These vegetation types are representative of variation in topo‐edaphic conditions. Herbaceous meadows tend to form on mineral soils of glacial or volcanic sediments as well as along shorelines and receive nutrients from these substrates. Wet meadows occupy saturated mineral or organic soils associated primarily with cold, wet conditions in interior Alaska (Ping et al. 2017); however, this term was also applied less frequently in coastal areas. Wet meadow habitat occurs at higher elevations in interior Alaska, merging with alpine habitat. Bog and fen habitats develop on peat soils and differ by greater fertility in fens due to groundwater nutrient exchange (Ping et al. 2017). Bog and muskeg are used interchangeably in this paper.

### 2.3 Data Collection and Processing

We accessed 175 *P. dilatata* herbarium records, including 168 records from ARCTOS (2023), University of Alaska Fairbanks, and seven additional records from E‐Flora BC, UBC, CA (Klinkenberg 2018). Among the ARCTOS records, 166 were from Alaska, and two were from adjacent Yukon Territory. Twenty‐nine of the ARCTOS Alaska records were excluded due to poor quality or absent images, leaving 139 records. The E‐Flora BC specimens provided three additional collections from the U.S. Aleutians as well as four collections from the Russian Commander Islands. The final data set of 146 records provided high‐resolution images of leaves, inflorescences, and floral organs. These records were collected between 1932 and 2017, with 50% collected between 1975 and 2000. These records are provided in Table A1 in Appendix 1. In this study, the herbarium sheet represents the sample; data from sheets containing multiple specimens were averaged to represent a single specimen sample. Latitude and longitude coordinates were accessed with images and were adjusted for precision using maps and aerial images.

Plants were measured using the measuring tool on GIMP2.10.34 (GIMP 2019). The metric scale in each scanned image was used to calibrate measurements. Floral metrics included lip length, spur length, lateral sepal width, and lateral sepal length; inflorescence metrics included inflorescence width, inflorescence length from lowermost to uppermost flower, and number of flowers; and leaf metrics included largest leaf width and largest leaf length. To avoid measuring immature floral organs, when possible, metrics were taken from flowers on the lower halves of inflorescences. Curved spurs were measured in segments, which were summed to total lengths. Dorsal petals and sepals and column dimensions could not be measured due to distortion caused by pressing and drying. For each plant, up to three measures of floral parts from different flowers were measured and averaged when available. However, in many cases, only single measures could be made.

Specimen drying often contracts floral organs (Parnell et al. 2013). To assess the amount of shrinkage in spur length and lip length, data from *N* = 20 herbarium sheets collected in Southeast Alaska were compared to measures taken from *N* = 26 fresh flowers collected in Southeast Alaska (data presented in Wallace and Bowles 2023). Percent shrinkage was calculated from mean values as [(fresh mean − dry mean)/fresh mean]*100, and significance of shrinkage was tested with ANOVA by comparing fresh versus dried lip and spur lengths.

### 2.4 Data Analysis

#### 2.4.1 How Is Phenotypic Variation Environmentally Structured Among Regions and Habitats and Their Effects on Climate and Edaphic Conditions?

To understand spatial structure at the level of individual traits, we first examined the distribution of all individual traits across the four regions and habitats described above. Their morphological variation was then reduced to orthogonal principal components using principal components analysis (PCA) with a correlation matrix. Trait factor loadings were examined to interpret how traits corresponded to the first and second PCA axes. The first PCA axis accounts for the greatest variance and acts as a size vector in morphometrics, while subsequent axes may be interpreted biologically (e.g., Berner 2011; Paušič et al. 2019). Data were log‐transformed for analysis on NCSS 9 Statistical Software (2013).

To address how phenotypic variation was environmentally structured, the first and second principal components were tested as continuous variables in an Analysis of Covariance General Linear Model with region and habitat as factors, and elevation and latitude as covariates, as high elevations and northern extremes tend to affect phenotype expression. Regions included the four geographic regions (Southeast Alaska, Interior Alaska, Southwest Alaska, and the Aleutian Islands) and four habitats (muskeg bog, fen, meadow, and wet meadow). Vague habitat descriptions on specimen labels were clarified to the extent possible by examining Google Earth (https://earth.google.com/) air photos and published descriptions and using field knowledge of habitats in the collection area. Nine specimens were excluded due to lack of precise information. Elevation was accessed from the WorldClim dataset (available at http://www.worldclim.org/) (Fick and Hijmans 2017; Harris et al. 2020) for each set of coordinates. Data were ln‐transformed for analysis.

To test the relationship between phenotypic and climatic variation, we correlated *N* = 19 climatic variables, as well as latitude and elevation, from each specimen collection point with the first and second PCA axes. Such variables can impose physiological constraints on species and therefore can affect their phenotypes, abundance, and distributions (O'Donnell and Ignizio 2012). Specimen coordinates were used to identify climatic variables from the WorldClim 1950 to 2000 database (Fick and Hijmans 2017, Harris et al. 2020). Climatic variables that were significantly correlated with PCA axes were also tested for variation among geographic regions using a Kruskal–Wallis one‐way ANOVA.

#### 2.4.2 Are Floral and Non‐Floral Traits Decoupled, With Reduced Phenotypic Correlation and Contrasting Responses to Abiotic Factors?

To determine how vegetative and floral traits covary, we first compared their Pearson correlation coefficients, the slopes of their linear regression equations, and probabilities that the slopes were not equal to zero. In these comparisons, sepal length and width, lip length, and spur length were treated as floral traits, while leaf and inflorescence length and width and number of flowers were treated as vegetative (or non‐floral) traits. A one‐way ANOVA was then used to test whether correlation coefficients and Ln‐transformed regression slopes differed among the floral traits, using the vegetative traits as replicates. Following Berg (1960) and Armbruster et al. (1999), we expected that spur length, which is assumed to be under strong pollinator‐mediated selection, would have a lower correlation and regression slope than floral traits under weaker selection from pollinators and less decoupled from vegetative growth.

To further learn whether spur length and vegetative traits are decoupled, we tested whether spur length and leaf size respond differently to abiotic factors in a two‐factor ANCOVA, with elevation as a covariate. Leaf size was calculated as an index represented by leaf length × width. If these traits differ in their responses to abiotic factors, we expected this to be revealed by the presence or absence of significant interactions between factors. For these tests, we compared edaphic and climatic variables, where a significant interaction in trait size would indicate that size in different habitats is not independent of the effects of climate. Habitat was used as the edaphic variable because it reflects substrate. Habitats included meadow (wet meadow and herbaceous meadow combined) and peatland (fen and muskeg combined). These habitats were combined to avoid low replication (*N* = 2) in some regions, and because they tend to merge at high elevation and high latitude. Region was used as a proxy for climate by comparing the moderate coastal climate of Southwest and Southeast Alaska to the more extreme continental climate of Interior Alaska. The Aleutian region was omitted due to a lack of habitat replicates. All values were Ln‐transformed if needed to achieve normality. We expected that plant leaf area would represent an interactive response to edaphic and climatic factors, while spur length would respond to regional climatic differences, which may affect pollinators, but not edaphic factors, which tend to be local conditions that do not affect pollinators.

#### 2.4.3 How Does the Observed Phenotypic Variation of *P. dilatata* in Alaska Correspond to Infraspecific Varietal Designations?

We addressed the potential for the presence of *P. dilatata* varieties in the data set in comparison to unpublished and published studies within Alaska, the lower US, and range‐wide. Alaska studies included three analyses. *N* = 34 plants in the current data set were annotated by Sheviak (2002) for the Flora North America section on *P. dilatata* . These plants were annotated as *P. dilatata* “var. *albiflora*” (*n* = 17 plants), as “var. dilatata?” (*N* = 11 plants), and as showing selection toward var. “*leucostachys*” (*N* = 6 plants). A second Alaska study of *N* = 26 populations in Southeast Alaska identified two groups that represent variation within var. *dilatata* (Wallace and Bowles 2023). The current study represents a range‐wide Alaska data set. Two lower USA studies are represented by Wallace (2003), which included 122 samples among 30 populations and represented all three *P. dilatata* varieties, and by Adhikari and Wallace (2014), which represented 88 samples among 24 populations, including a single Alaska specimen. This latter study did not differentiate between var. *albiflora* and *dilatata* but separated var. *leucostachy*s. The range‐wide study includes data summarized from specimen examination for the Flora North America section on *P. dilatata* by Sheviak (2002).

To compare Alaska phenotypes with designated varieties, we analyzed their distributions among regions and habitats using spur length and spur–lip length ratio as continuous variables in a GLM ANOVA with region and habitat as factors. These results were then directly compared to the dimensions of designated varieties. Mean spur lengths within the dimensions established for var. *albiflora* would represent that variety. Spur lengths within the dimensions established for var. *dilatata* would represent that variety, and those spur lengths within the dimensions established for var. *leucostachys* would represent that variety. Likewise, a spur–lip ratio of < 1.0 represents var. *albiflora*, a ratio near 1.0 represents var. *dilatata*, and a ratio > 1.0 represents var. *leucostachys* (Sheviak 2002). Reported statistics are from dried herbarium specimens or from flowers preserved in FAA in Wallace (2003) and fresh flowers in Wallace and Bowles (2023). Measurements representing dried data may be 12.5%–13.5% smaller (this study, see below).

## 3 Results

Dried plants from Southeast Alaska had 13.54% shorter lips (*F* = 16.03, *p* = 0.00025) and 12.56% shorter spurs (*F* = 21.79, *p* = 0.00003) than fresh material did. This difference does not affect the analysis of dried material in the current study, but it should be accounted for in comparison with fresh material.

### 3.1 How Is Phenotypic Variation Environmentally Structured Among Regions and Habitats and Their Effects on Climate and Edaphic Conditions?

Individual floral and vegetative traits varied significantly across regions and habitats (Table A2 in Appendix 1). Plants tended to have larger leaves and inflorescences and more flowers in coastal meadows of Southwest Alaska and were smaller in Interior Alaska. Floral traits (excluding nectar spurs) were larger in Southwest Alaska and the Aleutians. Nectar spurs were shorter in the Aleutians and Interior Alaska. Variation also differed among habitats. Floral traits (excluding nectar spurs) also tended to be larger in meadows. However, nectar spurs were longer in Muskeg habitat.

**FIGURE 2:**
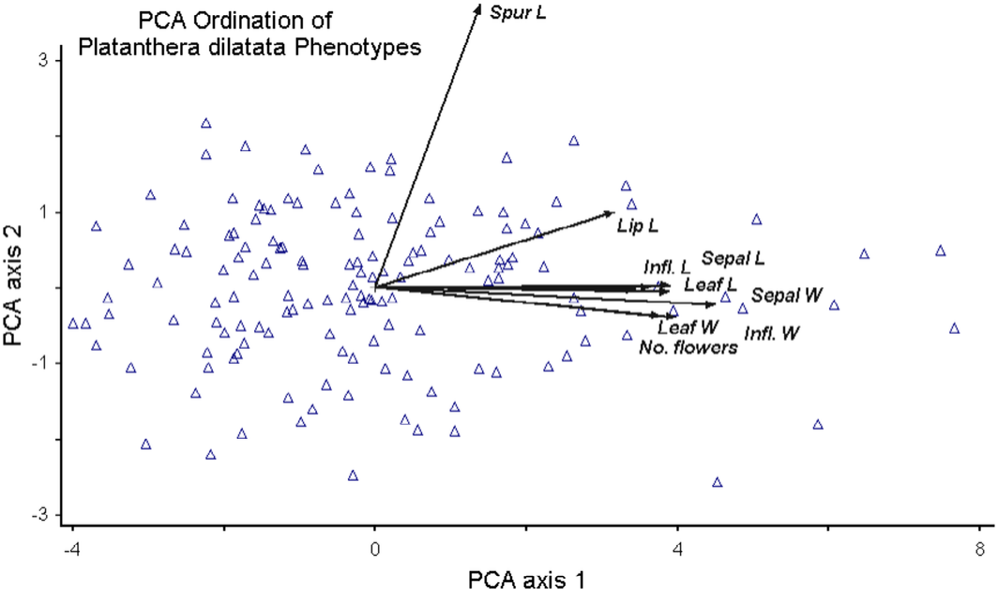
Principal components analysis ordination of herbarium samples based on floral and non‐floral trait values. Cumulative percent of variance extracted: axis 1 (60.094%), axis 2 (74.949%). Vectors represent trait factor loadings. See Table 1 for floral trait correlations with each axis.

When vegetative and floral traits were reduced to principal components (Figure 2), all traits were significantly (*p* < 0.0001) positively correlated with axis 1. Axis 2 was significantly negatively correlated with leaf width, inflorescence width and sepal width and length; and it was significantly positively correlated with inflorescence length, lip length, and spur length (Table 1). Spur length had the highest correlation (*r* ^2^ = 0.6177), which was twice the value for axis 1 (Table 1). Conversely, other significant traits were 3–4 times higher for axis 1.

**TABLE 1:** Pearson correlation coefficient (*R*), *R* ^2^, and probabilities of floral and vegetative trait correlations with PCA axis 1 and PCA axis 2.

Trait	Axis 1			Axis 2		
	*R*	*R* ^2^	*p*	*R*	*R* ^2^	*p*
No. flowers	0.7121	0.5072	**< 0.0001**	−0.0804	0.0065	0.3346
L Inflorescence	0.7209	0.5196	**< 0.0001**	0.2199	0.0483	**0.0077**
W Inflorescence	0.8497	0.7221	**< 0.0001**	−0.3262	0.1064	**0.0001**
L Leaf	0.6475	0.4192	**< 0.0001**	−0.0084	0.0001	0.9196
W Leaf	0.7052	0.4973	**< 0.0001**	−0.2028	0.0411	**0.0141**
L Lip	0.8059	0.6495	**< 0.0001**	0.0891	0.0079	0.2848
L Sepal	0.8485	0.7199	**< 0.0001**	−0.2099	0.0441	**0.0110**
W Sepal	0.8265	0.6831	**< 0.0001**	−0.2585	0.0668	**0.0016**
L Spur	0.5581	0.3115	**< 0.0001**	0.7859	0.6177	**< 0.0001**

*Note:* Probability values < 0.05 are in bold.

Abbreviations: L, length; No., number; W, width.

**FIGURE 3:**
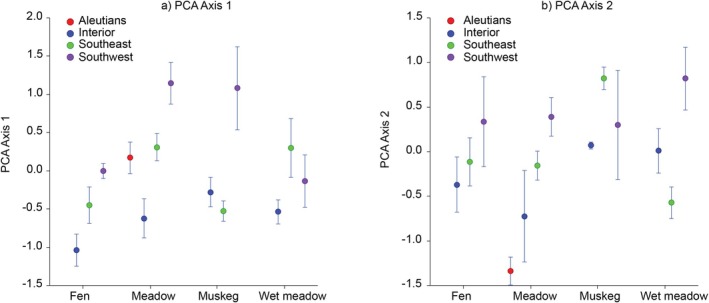
Mean (+SE) trait scores for PCA axis 1 (a) and axis 2 (b) scores in relation to region and habitat. ANCOVA: PCA 1 (Latitude *F* = 1.2, *p* = 0.263432; Elevation *F* = 7.14, *p* = 0.008581; Region *F* = 2.72, *p* = 0.047300; Habitat *F* = 0.87, *p* = 0.000261). PCA 2 (Latitude *F* = 0.11, *p* = 0.738185; Elevation *F* = 0.05, *p* = 0.816467; Region *F* = 3.32, *p* = 0.022366; Habitat *F* = 8.27, *p* = 0.000048).

Phenotypic variation, as shown by PCA axis 1 scores, was significantly related to distribution among regions and habitats, and elevation covaried with this distribution (Figure 3). Although there was no overall interaction between regions and habitats, Meadow and muskeg habitats in Southwest Alaska tended to have greater axis 1 scores, while most interior habitats tended to have the lowest axis 1 scores. Phenotypic variation, as shown by PCA axis 2 scores, was also significantly related to distribution among regions and habitats (Figure 3). Most Southwest Alaska phenotypes, as well as muskeg phenotypes in Southeast Alaska, had greater axis 2 scores. Aleutian meadows had extremely low axis 2 scores, and elevation covaried with this distribution. Neither latitude nor elevation covaried significantly with axis 2 scores.

Temperature‐related variables were the primary climatic factors corresponding significantly to the first and second PCA axes (Table 2). Axis 1, which represents vegetative and floral traits, was positively correlated with mean annual temperature, as well as mean temperatures of the coldest and driest quarters, and the minimum temperature of the coldest month. Axis 1 was negatively correlated with the annual temperature range and the mean diurnal temperature range, maximum temperature of the warmest month, and mean temperature of the warmest quarter, as well as temperature and precipitation seasonality. Axis 2, which most strongly represents nectar spur length, was positively correlated with mean annual temperature, mean temperature of the warmest quarter, and maximum temperature of the warmest month, as well as isothermality. Latitude was weakly, though significantly, negatively correlated with axis 1 (*r* ^2^ = 0.035, *p* = 0.0283) and not significantly correlated with axis 2 (*r* ^2^ = 0.006, *p* = 0.353). Elevation was significantly negatively correlated with axis 1 (*r* ^2^ = 0.087, *p* < 0.001) and not correlated with axis 2 (*r* ^2^ = 0.013, *p* = 0.190).

**TABLE 2:** Pearson correlation coefficients (*R*), *R* ^2^, and probabilities (p) for climatic correlations with PCA axis 1 and PCA axis 2 ordination of phenotypic groups.

Variables^a^	Axis 1			Axis 2		
	*R*	*R* ^2^	*p*	*R*	*R* ^2^	*p*
Min temp cold mo	0.2853	0.0814	**0.0005**	0.1033	0.0107	0.2149
Mean temp cold qtr	0.2764	0.0764	**0.0007**	0.1308	0.0171	0.1155
Mean ann temp	0.2242	0.0503	**0.0065**	0.2247	0.0505	**0.0064**
Mean temp driest qtr	0.1425	0.0203	0.0861	0.0746	0.0056	0.3709
Isothermality	0.0675	0.0046	0.4182	0.1605	0.0258	**0.0529**
Precip seasonality	−0.3508	0.1231	**< 0.0001**	0.0865	0.0075	0.2992
Ann temp range	−0.3089	0.0954	**0.0001**	−0.0073	0.0001	0.9301
Mean diur temp range	−0.3030	0.0918	**0.0002**	0.0413	0.0017	0.6203
Temp seasonality	−0.3000	0.0900	**0.0002**	−0.0210	0.0004	0.8014
Max temp warm mo	−0.2279	0.0519	**0.0057**	0.2820	0.0795	**0.0006**
Precip wett mo	−0.0948	0.0090	0.2553	0.1289	0.0166	0.1210
Precip wett qtr	−0.0869	0.0076	0.2969	0.1129	0.0127	0.1748
Mean temp warm qtr	−0.0679	0.0046	0.4157	0.3674	0.1350	**< 0.0001**
Precip warm qtr	−0.0616	0.0038	0.4598	0.0602	0.0036	0.4706
Ann precip	−0.0552	0.0030	0.5080	0.1078	0.0116	0.1953
Precip cold qtr	−0.0491	0.0024	0.5559	0.1117	0.0125	0.1796
Precip driest mo	−0.0293	0.0009	0.7254	0.1273	0.0162	0.1257
Mean temp wet qtr	−0.0289	0.0008	0.7295	0.1174	0.0138	0.1582
Precip driest qtr	−0.0074	0.0001	0.9295	0.1123	0.0126	0.1773

*Note:* Data are ranked by correlation coefficients for axis 1. Climatic data are from WorldClim data. Probability values < 0.05 are in bold.

^a^ Minimum temperature coldest month, Mean temperature coldest quarter, Mean annual temperature, Isothermality, Precipitation seasonality, Annual temperature range, Annual mean diurnal temperature range, Temperature seasonality, Maximum temperature warmest month, Precipitation wettest month, Precipitation wettest quarter, Mean temperature warmest quarter, Precipitation warmest quarter, Annual precipitation, Precipitation coldest quarter, Precipitation driest month, Mean temperature wettest quarter, Precipitation driest quarter.

Climatic variables also varied significantly among physiographic regions (Table A3 in Appendix 1). In comparison to most other regions, the Interior region had a significantly lower mean annual temperature, as well as a lower minimum temperature of the coldest month and a lower mean temperature of the coldest quarter. Conversely, the interior region had greater diurnal and annual temperature ranges and greater mean temperature of the warmest month; it also had a greater temperature and precipitation seasonality. The Aleutian region had the lowest mean annual and diurnal temperature ranges, lower maximum and mean temperatures, lower temperature seasonality, and higher minimum temperature of the coldest month.

Across all habitats, elevation was significantly positively correlated with 16 climatic variables, including colder temperatures and lower precipitation as well as greater precipitation and temperature seasonality and greater diurnal and annual temperature ranges (Table A4 in Appendix 1). Temperature‐related *r* ^2^ values averaged 0.32 (0.35 SE) and were 400% higher than precipitation *r* ^2^ values, which averaged 0.08 (0.01 SE).

### 3.2 Are Floral and Non‐Floral Traits Decoupled, With Reduced Phenotypic Correlation and Contrasting Responses to Abiotic Factors?

All floral and non‐floral traits were significantly correlated (Table 3). However, the correlations and slopes of floral traits regressed against non‐floral traits differed significantly. Nectar spur length correlations were lower than other floral traits, while slopes were lower for spur length than for sepal width and intermediate for sepal and lip lengths (Table 3).

**TABLE 3:** Correlation (Corr.), probabilities (Prob), and regression slopes (Slope) of comparisons between floral and non‐floral traits.

Trait	Statistic	No. Flowers	W Leaf	L Leaf	W Inflorescence	L Inflorescence	Mean	SE
L Sepal	Corr	0.5184	0.5903	0.5476	0.6933	0.4862	0.5672a	0.0359
	Prob	< 0.0001	< 0.0001	< 0.0001	< 0.0001	< 0.0001		
	Slope	6.0034	3.1157	1.8652	2.7840	1.4162	3.0369ab	0.8022
W Sepal	Corr	0.6142	0.6431	0.4548	0.7312	0.5430	0.5973a	0.0467
	Prob	< 0.0001	< 0.0001	< 0.0001	< 0.0001	< 0.0001		
	Slope	15.3031	7.3016	3.3319	6.3156	3.4010	7.1306b	2.1889
L Lip	Corr	0.4063	0.4847	0.4497	0.5907	0.4355	0.4734a	0.0319
	Prob	< 0.0001	< 0.0001	< 0.0001	< 0.0001	< 0.0001		
	Slope	4.8304	2.6254	1.5719	2.4341	1.3018	2.5527a	0.6219
L Spur	Corr	0.2638	0.2121	0.3040	0.2360	0.4554	0.2943b	0.0431
	Prob	0.0018	0.0128	0.0003	0.0055	< 0.0001		
	Slope	2.4135	0.8850	0.8182	0.7486	1.0480	1.1827a	0.3117

*Note:* Floral trait correlation means ANOVA: *F* = 11.74, *p* = 0.0003. Floral trait slope means ANOVA: *F* = 7.57, *p* = 0.0023. Mean correlations or slopes sharing different lowercase letters differ with the Tukey–Kramer Multiple‐Comparison Test at *p* < 0.05.

Abbreviations: L, length; No., number; W, width.

**FIGURE 4:**
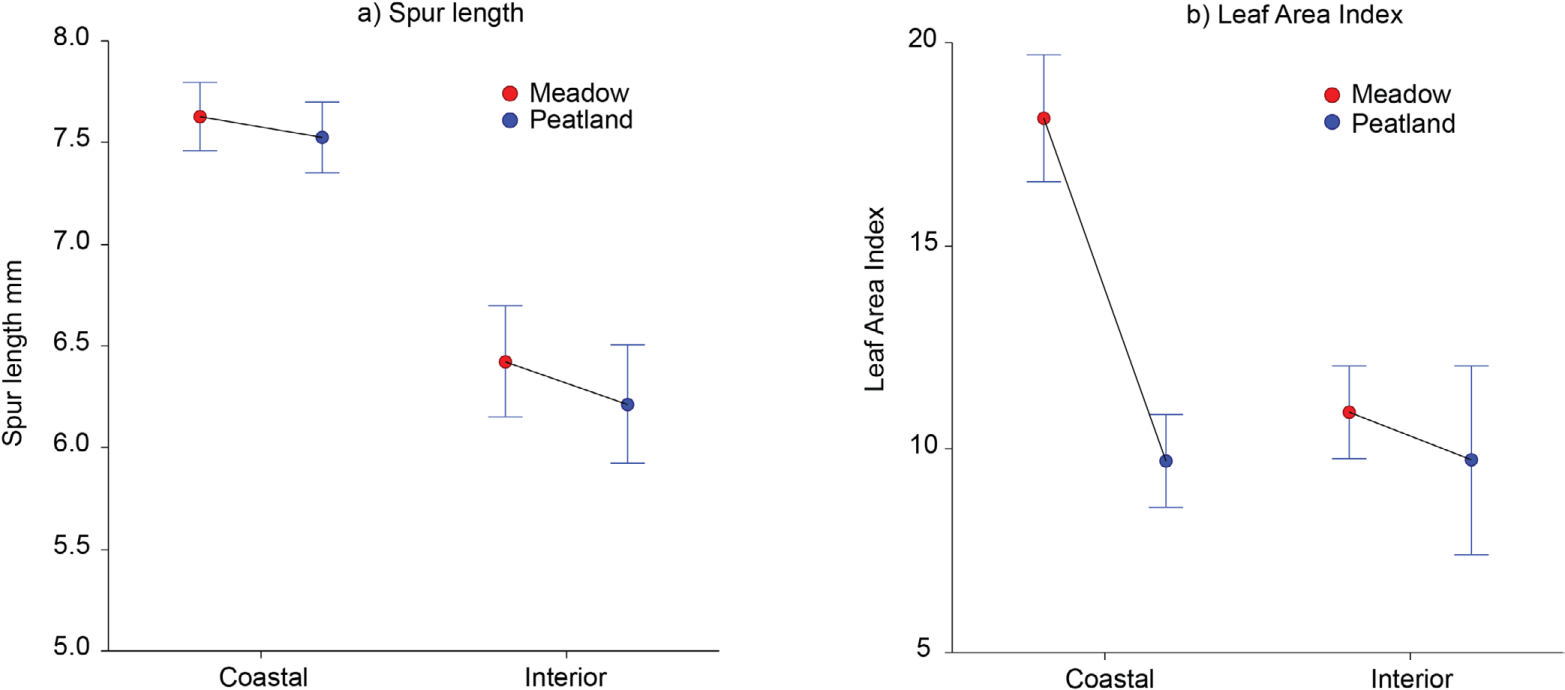
Differences in mean (+SE) nectar spur length (a) and leaf area index (b) between meadow and peatland habitats in coastal and interior regions of Alaska. ANCOVA: Spur length (Elevation *F* = 5.98, *p* = 0.015964; Region *F* = 10.64, *p* = 0.001447; Habitat *F* = 0.31, *p* = 0.576188, Region × Habitat *F* < 0.001, *p* = 0.955933). Leaf area index (Elevation *F* = 9.05, *p* = 0.003211; Region *F* = 0.01, *p* = 0.904847; Habitat *F* = 7.07, *p* = 0.008912; Region × Habitat *F* = 3.73, *p* = 0.055731).

Analysis of covariance comparing spur length with leaf area revealed significant region and habitat differences, with a significant region × habitat interaction for leaf area, but not for spur length, as well as significant covariance with elevation (Figure 4). Leaf area was greater in coastal meadow than in peatland habitat, but not so in interior habitats. In contrast, spur length was shorter in the interior region in either meadow or peatland habitats. Elevation was a significant covariate. It was highest in Interior Alaska habitats, intermediate in Southeast Alaska meadows, and lower in all other habitats (data not shown).

### 3.3 How Does the Observed Phenotypic Variation of *P. dilatata* in Alaska Correspond to Infraspecific Varietal Designations?

**FIGURE 5:**
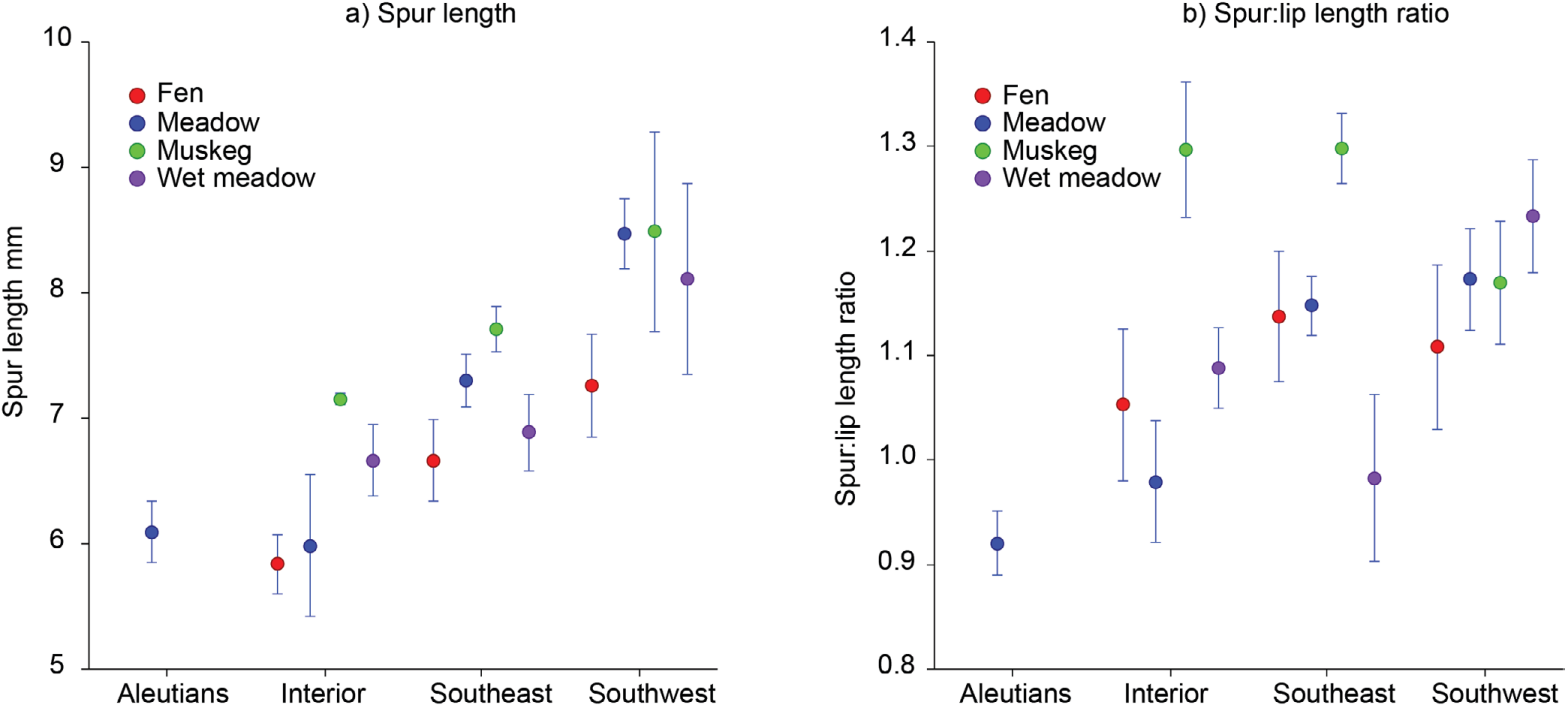
Mean (+SE) *Platanthera dilatata* nectar spur length (a) and spur–lip length ratio (b) in relation to region and habitat in Alaska. ANOVA: Spur length (Region *F* = 6.45, *p* = 0.000434; Habitat *F* = 2.80, *p* = 0.042690; Region × Habitat *F* = 0.41, *p* = 0.926828), spur–Lip length ratio (Region *F* = 4.29, *p* = 0.006509; Habitat *F* = 7.25, *p* = 0.000163; Region × Habitat *F* = 1.24, *p* = 0.279083).

Both nectar spur length and nectar spur–lip length ratio varied significantly among regions and habitats (Figure 5). Nectar spur lengths ranged from shorter spurs averaging about 6 mm in the Aleutians and Interior Alaska fens and meadows to longer spurs averaging 8–9 mm in Southwest Alaska meadows and muskeg. Intermediate spur lengths averaging about > 6 to < 8 mm occurred in Southeast Alaska, as well as in interior wet meadows and southwest fens. The nectar spur–lip length ratio averaged < 0.1 in the Aleutians, 1.3 in muskeg habitat in Interior and Southeast Alaska, and 1–1.2 elsewhere.

## 4 Discussion

Despite the assumption that selection by pollinators and other biotic agents strongly affects floral variation, abiotic factors are increasingly recognized to have an influence on floral and vegetative traits (Arista et al. 2013; Caruso et al. 2018, 2020; Sletvold 2019). Our data analysis of a widespread species that occupies geographically divergent habitats strongly supports this contention. These data suggest that climatic and edaphic variables may be responsible for both floral and vegetative traits that comprise substantial intraspecific variation and jointly determine integrated phenotypic expression. In Alaska, *P. dilatata* is represented by a continuum of phenotypes of plants exhibiting either large or small leaves, inflorescences, and flowers, each of which is also represented by plants with either long or short nectar spurs. Significant correlations of the former traits, but not nectar spur length, with climate variables indicate that contrasting coastal and continental climates drive this phenotypic variation. Large plants are associated with a moderate coastal climate, and small plants are associated with a more extreme continental climate. Although large and small phenotypes also partition among habitats, the significant interaction between region and habitat indicates that plant size is not independent of climate. By contrast, nectar spur length, which is frequently influenced by pollinator‐mediated selection (Fenster et al. 2004), is poorly correlated with leaf, flower, and inflorescence size in *P. dilatata* in Alaska (Table 3) and correlated with few climatic variables as the predominant variable associated with PCA axis 2 (Table 2). These results suggest that the traits of *P. dilatata* have contrasting forces influencing their expression that operate locally and regionally across its geographic range. Given the continuum of phenotypic variation demonstrated by *P. dilatata* in Alaska, it is difficult to differentiate varieties by spur–lip length relationships, as suggested by the current infraspecific taxonomy.

### 4.1 How Is Phenotypic Variation Environmentally Structured Among Regions and Habitats and Their Effects on Climate and Edaphic Conditions?

Depending upon environmental context, the regional presence of distinct phenotypes represents a complex interaction involving both abiotic and biotic selection, and the latter may vary between selection by pollinators and other biotic agents that affect reproduction and survivorship (Caruso et al. 2018; Sletvold 2019). As with other plant species, the distributions of north temperate terrestrial orchids usually correspond to climate‐induced migration (e.g., Pfeifer, Wiegand, et al. 2006; Pfeifer, Heinrich, et al. 2006) and interactions with variation in soil chemistry and fertility (e.g., Wherry 1918; Sheviak 1974; Bowles et al. 2005, 2021; Tsiftsis et al. 2012; Djordjević and Tsiftsis 2020). The correspondence of distinct *P. dilatata* phenotypes to different regions and habitats in Alaska, and the region × habitat interaction shown between interior and coastal Alaska regions, suggests that phenotypic responses occur at the regional scale in relation to climatic variation, but that edaphic effects may determine local phenotypes under moderate, but not extreme, climate conditions.

Large‐flowered plants, which have larger leaves and inflorescences, have a primarily coastal distribution in Southwest and Southeast Alaska (Figure 3a; Table A2 in Appendix 1) that is associated with a marine‐moderated climate characterized by cool summers, warm winters, and low temperature and precipitation seasonality, conditions likely to result in low physiological constraints (O'Donnell and Ignizio 2012). In contrast, small‐flowered plants, which have smaller leaves and inflorescences, are associated with continental climatic conditions in interior Alaska (Figure 3a; Table A2 in Appendix 1), characterized by warmer summer and colder winter temperatures, greater diurnal temperature ranges, and greater climatic seasonality, conditions more likely to induce greater overall physiological constraints that may limit species growth and distribution (O'Donnell and Ignizio 2012).

At the local scale, topo‐edaphic factors appear to affect phenotypic expression. Large plants tend to occur more frequently in meadow habitats that are disassociated from more strongly acidic peatlands with low fertility (Wallace and Bowles 2023), and greater soil fertility may contribute to their larger leaves (Ordoñez et al. 2009). However, climate appears to regulate this effect, as larger plants appear to be restricted to coastal climates. In contrast, smaller plants occupy meadow habitats in a continental climate, as well as peatlands in either coastal or continental climates (Wallace and Bowles 2023). The negative relationship between elevation and most plant traits may represent an increasing stress gradient. Alpine habitats are well known for stress that causes smaller vegetation (e.g., Callaway et al. 2002). Climatic data suggest that such stress at higher elevations would be related to lower temperature and precipitation (as well as greater precipitation seasonality) and greater diurnal and annual temperature ranges. Temperature extremes may cause greater stress than precipitation extremes, as indicated by the higher correlations between elevation and temperature variables than with precipitation variables.

Likewise, small phenotypes account for greater representation in interior Alaska, where wet meadow habitats in cold, wet conditions could limit nutrient availability and plant growth more than in southern populations (e.g., Edwards et al. 2006). Colder temperatures and greater climatic extremes associated with northern latitudes are also known to limit plant distribution (Normand et al. 2009) as well, and small‐flowered phenotypes may characterize populations of *P. dilatata* at its northern range limit.

### 4.2 Are Floral and Non‐Floral Traits Decoupled, With Reduced Phenotypic Correlation and Contrasting Responses to Abiotic Factors?

The integration of traits into a complex phenotype is controlled by external forces acting within a genetically controlled developmental framework (Murren 2002, 2012). Because this occurs within ongoing spatial and temporal variation in abiotic and biotic factors, substantial phenotypic variation can be manifested within species, as we have demonstrated for *P. dilatata* in Alaska. Berg (1960) hypothesized that floral and vegetative traits may be decoupled to maximize pollen transfer with interacting pollinators. Thus, floral traits may be expected to respond to pollinator pressure, whereas vegetative traits should more closely align with abiotic factors. While some studies have supported this prediction, others have found that floral traits respond to abiotic factors in a similar manner to vegetative traits (reviewed in Pélabon et al. 2013).

Pollination of *Platanthera* flowers can be considered specialized since pollinators must probe the spur to effectively pollinate flowers (Hapeman and Inoue 1997; Plendi et al. 2024). Many *Platanthera* species pairs are isolated from one another by how pollinia are placed on pollinators (e.g., Boberg et al. 2014), and the nectar spur plays a large role in partitioning pollinators by tongue length. Thus, nectar spur length in *Platanthera* is expected to be under stronger selection by pollinators (Hapeman and Inoue 1997; Fenster et al. 2004) than by abiotic factors. Although pollinators can select multiple floral traits, spur length controls pollinator efficiency through the positioning of pollinia, as well as controlling access to the food reward, both key factors that would cause a strong response by nectar spurs to how pollinators interact with the flowers (Sletvold 2019). Accordingly, we suggest that nectar spur length is decoupled from other floral and vegetative traits in *P. dilatata* because it responds to different external factors. Diversifying selection on nectar spur length within *P. dilatata* could be driven by the numerous insects that have been documented as pollinators across its geographic range. For example, in Southeast Alaska, Noctuidae moths (Bowles and Armstrong 2021; Wallace and Bowles 2023), whose tongue lengths average < 11 mm but can reach 18 mm (Little et al. 2005; Zenker et al. 2011; Zhang et al. 2021), are primary pollinators, but the Sphingidae hawkmoth *Hyles gallii* , whose tongue length may exceed twice the spur length of *P. dilatata* , appears to be a secondary pollinator in this area and could select for longer spur lengths (Bowles and Armstrong 2021; Wallace and Bowles 2023). This sphingid also occurs in interior Alaska but appears to be absent from the Aleutian Islands and Southwest Alaska (iNaturalist unpublished data; University of Alaska Museum of the North unpublished data accessed on ARCTOS). Its absence from the Aleutians could be a factor in the presence of only short‐spurred phenotypes in that region. However, plants in the Southwest Alaska Peninsula have longer nectar spurs despite the apparent absence of hawkmoths. In other geographic areas, documented pollinators have included a Hesperiidae butterfly in Newfoundland, Canada (Boland 1993), and *Bombus* bumblebees and a Nymphalidae butterfly in British Columbia, Canada (van der Voort et al. 2022). Thus, if the primary pollinator types vary in abundance across populations of *P. dilatata* in Alaska, they may drive variation in nectar spur length.

### 4.3 How Does the Observed Phenotypic Variation of *P. dilatata* in Alaska Correspond to Infraspecific Varietal Designations?

The infraspecific taxonomy of *P. dilatata* includes three varieties that are distinguished by spur length or by the ratio of spur length to lip length (Luer 1975; Sheviak 2002). These treatments quantify var. *albiflora* spur lengths as usually 2–8 mm in length, var. *dilatata* spur lengths as usually > 4–10 mm, and var. *leucostachys* spur lengths as usually > 8–20 mm (reviewed in Wallace and Bowles 2023). Our nectar spur length data support the suggestions of Sheviak (2002) and Wallace and Bowles (2023) that plants consistent with the descriptions for *P. dilatata* vars. *albiflora* and *dilatata* occur frequently in Alaska. Marginally longer spur lengths suggest that selection toward var. *leucostachys* may be occurring in a few populations. Our spur–lip length ratio data also suggest that var. *leucostachys* is absent, and the few records of extreme ratios that would be consistent with var. *leucostachys* may be based more on shorter lip lengths than longer spur lengths. However, the continuous variation that was observed here and reported in other studies of *P. dilatata* (e.g., Adhikari and Wallace 2014) is problematic for varietal identification with the current taxonomy. Instead, we have found that phenotypic variation in *P. dilatata* in Alaska is more complex than the taxonomic assignment of plants to varieties based on nectar spur length and its relationship to lip length.

Whereas it has previously been assumed that pollinator‐mediated selection is the main driver of taxonomically recognized groups within this species, our results suggest this may be incorrect and that floral traits may not function as a modular unit. We suggest that climatic and edaphic factors drive phenotypic differences in flower and plant size, and that such variation responds to geographic region as well as habitat differences in Alaska. Whether these differences have been repeatedly established after independent colonization of areas across Alaska or stem from historically isolated groups is still unclear, but genetic differences that also correspond to phenotypically differentiated populations in Southeast Alaska (Wallace and Bowles 2023) suggest a lesser role for plasticity of these traits. Because it is unknown which plant traits have fixed genetic differences, taxonomic interpretations of the phenotypic variation are also not clear. If lip length and spur length vary independently across the landscape, then expressing taxonomic differences based on the relationship between these traits is questionable.

## 5 Conclusions

Variation in vegetative traits and floral traits that are not directly involved in pollination were found to associate with variation in climate and edaphic features, suggesting a stronger role for abiotic factors to influence these traits. Abiotic variation is expected to be dynamic among environments, thus shaping climate‐edaphic interactions that ultimately lead to complex phenotypes, such as the large and small plants of *P. dilatata* . Under a moderate climate, fertile habitats foster large plants, while in more stressful habitats, plants tend to be smaller. Small plants also occur under more extreme climates, regardless of habitat, indicating that edaphic effects are not independent of climate. Independent of the size of the plant, nectar spur length can be longer or shorter because it responds to different factors, presumably related to local pollinators, across habitats and regions. Origins of distinct phenotypes may include (1) habitat selection for differentiation in plant sizes linked with different flower sizes, exclusive of nectar spur length, or (2) migration and immigration of historic lineages genetically adapted to different habitat conditions. We cannot differentiate these mechanisms with the current dataset, and future studies that evaluate genetic structure and the history of *P. dilatata* in Alaska would clarify whether phenotypically similar populations have shared ancestry or if common traits arose independently in isolated areas, suggesting a role for plasticity in these traits. Given the continuum of variation, it is difficult to assign plants to discrete taxonomic groups in Alaska, and the decoupling of spur and lip length reported in this study makes their use problematic as diagnostic traits of the varieties. If the variation reported for *P. dilatata* in Alaska has a similar pattern across North America, it will warrant a more detailed review of taxonomic relationships across the entire geographic range.

## Author Contributions

**Marlin L. Bowles:** conceptualization (lead), data curation (lead), formal analysis (lead), investigation (equal), methodology (lead), project administration (lead), visualization (lead), writing – original draft (lead), writing – review and editing (equal). **Lisa E. Wallace:** conceptualization (supporting), formal analysis (supporting), funding acquisition (lead), investigation (equal), methodology (supporting), visualization (supporting), writing – review and editing (equal).

## Conflicts of Interest

The authors declare no conflicts of interest.

## Supporting information


Data S1.

## Data Availability

All the required data are uploaded as Supporting Information.

## Appendix 1

**TABLE A1:** Catalog numbers of accessed specimens.

Source	Herb.	No.	Source	Herb.	No.	Source	Herb.	No.
ARCTOS	ALA	3508	ARCTOS	ALA	42491	ARCTOS	ALA	57955
ARCTOS	ALA	4060	ARCTOS	ALA	42492	ARCTOS	ALA	58007
ARCTOS	ALA	4194	ARCTOS	ALA	42753	ARCTOS	ALA	58301
ARCTOS	ALA	4330	ARCTOS	ALA	43166	ARCTOS	ALA	58457
ARCTOS	ALA	4734	ARCTOS	ALA	43428	ARCTOS	ALA	58476
ARCTOS	ALA	5397	ARCTOS	ALA	43429	ARCTOS	ALA	58834
ARCTOS	ALA	5903	ARCTOS	ALA	44215	ARCTOS	ALA	60129
ARCTOS	ALA	6021	ARCTOS	ALA	44216	ARCTOS	ALA	60493
ARCTOS	ALA	6033	ARCTOS	ALA	44217	ARCTOS	ALA	61873
ARCTOS	ALA	6316	ARCTOS	ALA	44218	ARCTOS	ALA	61879
ARCTOS	ALA	6885	ARCTOS	ALA	44219	ARCTOS	ALA	62883
ARCTOS	ALA	8761	ARCTOS	ALA	44220	ARCTOS	ALA	63101
ARCTOS	ALA	9840	ARCTOS	ALA	44222	ARCTOS	ALA	63867
ARCTOS	ALA	9999	ARCTOS	ALA	44223	ARCTOS	ALA	66943
ARCTOS	ALA	10093	ARCTOS	ALA	44224	ARCTOS	ALA	66965
ARCTOS	ALA	11481	ARCTOS	ALA	44225	ARCTOS	ALA	67280
ARCTOS	ALA	11665	ARCTOS	ALA	44906	ARCTOS	ALA	67311
ARCTOS	ALA	11813	ARCTOS	ALA	44907	ARCTOS	ALA	71803
ARCTOS	ALA	12391	ARCTOS	ALA	45439	ARCTOS	ALA	75228
ARCTOS	ALA	12778	ARCTOS	ALA	45440	ARCTOS	ALA	81884
ARCTOS	ALA	15975	ARCTOS	ALA	45441	ARCTOS	ALA	81885
ARCTOS	ALA	20919	ARCTOS	ALA	46419	ARCTOS	ALA	82145
ARCTOS	ALA	26346	ARCTOS	ALA	46422	ARCTOS	ALA	84424
ARCTOS	ALA	26353	ARCTOS	ALA	46423	ARCTOS	ALA	141179
ARCTOS	ALA	26355	ARCTOS	ALA	46424	ARCTOS	ALA	141620
ARCTOS	ALA	26356	ARCTOS	ALA	46425	ARCTOS	ALA	144441
ARCTOS	ALA	26357	ARCTOS	ALA	47195	ARCTOS	ALA	144907
ARCTOS	ALA	26358	ARCTOS	ALA	48291	ARCTOS	ALA	145339
ARCTOS	ALA	26361	ARCTOS	ALA	48913	ARCTOS	ALA	145785
ARCTOS	ALA	26362	ARCTOS	ALA	50643	ARCTOS	ALA	146606
ARCTOS	ALA	26363	ARCTOS	ALA	50644	ARCTOS	ALA	147821
ARCTOS	ALA	26364	ARCTOS	ALA	50645	ARCTOS	ALA	147950
ARCTOS	ALA	26365	ARCTOS	ALA	50646	ARCTOS	ALA	153533
ARCTOS	ALA	26366	ARCTOS	ALA	50877	ARCTOS	ALA	153534
ARCTOS	ALA	26402	ARCTOS	ALA	50919	ARCTOS	ALA	240841
ARCTOS	ALA	31330	ARCTOS	ALA	51098	ARCTOS	ALA	240939
ARCTOS	ALA	38338	ARCTOS	ALA	51099	ARCTOS	ALA	248484
ARCTOS	ALA	38419	ARCTOS	ALA	51100	ARCTOS	ALA	1148488
ARCTOS	ALA	39081	ARCTOS	ALA	51101	ARCTOS	ALA	1148506
ARCTOS	ALA	41270	ARCTOS	ALA	51103	UBC	ASC	17469
ARCTOS	ALA	41280	ARCTOS	ALA	51104	UBC	MW	48075
ARCTOS	ALA	41336	ARCTOS	ALA	51118	UBC	MW	48076
ARCTOS	ALA	41645	ARCTOS	ALA	51243	UBC	MW	156292
ARCTOS	ALA	41891	ARCTOS	ALA	51381	UBC	MW	163402
ARCTOS	ALA	41892	ARCTOS	ALA	52660	UBC	NY	4119624
ARCTOS	ALA	41893	ARCTOS	ALA	52661	UBC	NY	4120040
ARCTOS	ALA	41894	ARCTOS	ALA	52662			
ARCTOS	ALA	42118	ARCTOS	ALA	52663			
ARCTOS	ALA	42194	ARCTOS	ALA	54568			
ARCTOS	ALA	42490	ARCTOS	ALA	56461			

Abbreviations: ARCTOS. ALA, University of Alaska; MW, Moscow State University; NY, New York Botanic Garden; UBC. ASC, Arizona State University.

**TABLE A2:** Mean (+SE) floral and non‐floral trait variation in *Platanthera dilatata* across regions and habitats in Alaska.

Trait		Habitat				Region				ANOVA		
		Fen	Meadow	Muskeg	Wet meadow	Aleutians	Interior	Southeast	Southwest	Factor	*F* ratio	*p*
	*N*	18	67	35	17	14	24	75	24			
No. Flowers	Mean	18.1220	24.9677	15.5835	13.5311	4.8434	17.8674	21.7700	27.7236	Habitat	3.53	0.0169
	SE	2.6466	1.3718	1.8980	2.7233	3.0010	2.2920	1.2966	2.2920	Region	3.12	0.0287
W Leaf mm	Mean	11.3781	15.1257	9.1311	13.4097	10.4805	11.3880	10.8260	16.3501	Habitat	15.66	< 0.0001
	SE	0.9874	0.5118	0.7081	1.0161	1.1196	0.8551	0.4837	0.8551	Region	5.85	0.0009
L Leaf cm	Mean	8.8627	10.2913	7.9147	8.7487	4.8061	9.2430	9.4976	12.2708	Habitat	2.37	0.0738
	SE	0.7828	0.4057	0.5614	0.8055	0.8876	0.6779	0.3835	0.6779	Region	2.97	0.0347
W Inflorescence cm	Mean	6.5760	7.7482	6.1295	5.3520	2.6291	6.8093	7.4196	8.9476	Habitat	1.88	0.1371
	SE	0.6500	0.3369	0.4661	0.6688	0.7370	0.5629	0.3184	0.5629	Region	3.09	0.0298
L Inflorescence mm	Mean	17.2560	19.8104	15.9755	19.2396	19.1769	16.3385	17.3793	19.3867	Habitat	7.27	0.0002
	SE	0.8193	0.4247	0.5876	0.8431	0.9290	0.7095	0.4014	0.7095	Region	2.41	0.0702
W Sep mm	Mean	2.1218	2.4987	1.9933	2.3881	2.0221	2.0645	2.3582	2.5571	Habitat	9.86	0.0000
	SE	0.0900	0.0467	0.0646	0.0927	0.1021	0.0780	0.0441	0.0780	Region	4.99	0.0027
L Sep mm	Mean	5.9566	6.6377	5.7665	7.1707	6.6207	5.9963	6.3294	6.5850	Habitat	6.66	0.0003
	SE	0.2153	0.1116	0.1544	0.2216	0.2442	0.1865	0.1055	0.1865	Region	1.05	0.3716
L Spur mm	Mean	6.3246	6.9613	7.3704	6.5467	5.5727	6.4098	7.1401	8.0804	Habitat	2.80	0.0427
	SE	0.2534	0.1314	0.1817	0.2608	0.2874	0.2195	0.1242	0.2195	Region	6.45	0.0004
L Lip mm	Mean	6.1528	6.6209	6.2073	7.3557	7.1942	5.8728	6.3663	6.9035	Habitat	2.58	0.0565
	SE	0.2162	0.1121	0.1551	0.2225	0.2452	0.1872	0.1059	0.1872	Region	4.11	0.0082

*Note:* ANOVA interactions are not significant and omitted. Standard errors (SE) are estimates for ANOVA.

Abbreviations: L, length; No., number; W, width.

**TABLE A3:** Relationship between median (+ inter quartile range) climatic variables and geographic regions.

Climate variables^a^	Aleutians			Interior			Southeast			Southwest			*χ* ^2^	*p*
	Median	*Z*	IQR	Median	*Z*	IQR	Median	*Z*	IQR	Median	*Z*	IQR		
Mean ann temp	3.77	a	1.52	−1.19	b	2.31	4.54	a	2.37	3.55	a	2.59	50.33	< 0.0001
Mean diur temp range	4.68	a	1.43	9.8	b	1.31	6.79	C	1.25	6.19	c	1.51	71.78	< 0.0001
Isothermality	27.19	ab	9.01	27.51	a	1.83	28.95	b	2.16	29.47	b	4.86	11.26	0.0104
Temp seasonality	419.96	a	131.39	957.96	b	112.38	627.55	c	141.47	560.12	c	148.74	62.84	< 0.0001
Max temp warm mo	12.81	a	1.53	18.05	b	2.25	16.8	c	1.9	15.25	d	1.92	64.16	< 0.0001
Min temp cold mo	−3.2	a	3.24	−17.5	b	4.65	−6.3	ac	5.55	−6.11	ac	5.02	49.82	< 0.0001
Ann temp range	17.1	a	4	35.5	b	4.7	23.1	c	5.45	20.95	c	4.98	65.46	< 0.0001
Mean temp driest qtr	6.71	a	4.58	−2.66	c	3.32	8.57	ad	4.58	−0.36	af	13.13	48.41	< 0.0001
Mean temp warm qtr	9.26	a	0.68	11.3	b	2.16	12.03	b	1.93	10.96	bc	1.07	47.22	< 0.0001
Mean temp cold qtr	−0.56	a	3.13	−11.93	b	4.08	−2.85	c	4.61	−2.64	c	3.65	47.33	< 0.0001
Precip seasonality	29.91	a	8.79	48.56	b	8.95	37.02	c	5.15	26.21	ad	11.88	62.45	< 0.0001

*Note:* Chi‐square probabilities are Kruskal–Wallis one‐way ANOVA on ranks corrected for ties. *Z* indicates Kruskal–Wallis multiple‐comparison *Z*‐value test, where medians with similar lowercase letters are not different with a Bonferroni adjustment.

^a^ Mean annual temperature, Mean diurnal temperature range, Temperature seasonality, Maximum temperature of the warmest month, Minimum temperature of the coldest month, Annual temperature range, Mean temperature of the driest quarter, Mean temperature of the warmest quarter, Mean temperature of the coldest quarter, Precipitation seasonality.

**TABLE A4:** Pearson correlation coefficients (*R*), *R* ^2^, and probabilities (p) for climatic correlations with elevation.

Variables^a^	*R*	*R* ^2^	*p*
Annual precip	−0.2704	0.0731	**0.0001**
Precip coldest qrtr	−0.2627	0.069	**0.0014**
Precip driest mo	−0.3142	0.0987	**0.0001**
Precip driest qrtr	−0.3265	0.1066	**0.0001**
Precip warmest qrtr	−0.1681	0.0282	**0.0426**
Precip wettest mo	−0.2439	0.0595	**0.0003**
Precip wettest qrtr	−0.2458	0.0604	**0.0028**
Precip seasonality	0.3612	0.1305	**< 0.0001**
Annual mean temp	−0.7046	0.4965	**< 0.0001**
Annual temp range	0.5444	0.2964	**< 0.0001**
Mean diurnal temp range	0.5395	0.2911	**< 0.0001**
Mean temp coldest qrtr	−0.6212	0.3859	**< 0.0001**
Mean temp driest mo	−0.4946	0.2446	**< 0.0001**
Mean temp warmest qrtr	−0.4276	0.1828	**< 0.0001**
Minimum temp coldest mo	−0.6248	0.3903	**< 0.0001**
Temp seasonality	0.5203	0.2707	**< 0.0001**

*Note:* Significant probabilities (p < 0.05) are indicated in bold.

^a^ Annual precipitation, Precipitation of the coldest quarter, Precipitation of the driest month, Precipitation of the driest quarter, Precipitation of the warmest quarter, Precipitation of the wettest month, Precipitation of the wettest quarter, Precipitation seasonality, Annual mean temperature, Annual temperature range, Mean diurnal temperature range, Mean temperature of the coldest quarter, Mean temperature of the driest month, Mean temperature of the warmest quarter, Minimum temperature of the coldest month, Temperature seasonality.
